# WELFARE REGIME TYPE AND ITS INFLUENCE ON THE FINANCIAL SECURITY OF FEMALE CAREGIVERS IN LATER LIFE

**DOI:** 10.1093/geroni/igad104.0178

**Published:** 2023-12-21

**Authors:** Mengya Wang

**Affiliations:** Iowa State University, Ames, Iowa, United States

## Abstract

Guided by life course theory, this paper examines the effect of early caregiving experience and later life-changing events on the financial security of older women using cross-national data from two panels (2004 and 2014) of the Survey of Health Aging and Retirement in Europe Analyses found that caregiving in midlife and subsequent life-changing events (being divorced or widowhood, changing employment status, and/or declining health) significantly affected women’s economic status in later life, Importantly, we found that early caregiving experience intensifies the effects of negative life events, and in turn further deteriorates women’s economic security in later life. Tests of welfare regime differences found that women who experienced a marital change (becoming divorced or widowed) in the dual breadwinner model were less likely to experience economic deprivation compared to the other two models. This finding is likely attributable to more gender equality in these countries which makes women more financially independent. Unfortunately, a significant association between welfare regime and caregiving was not found in this study. This study adds to the knowledge about the political, economic and cultural forces that shape the financial security of women in later life. Our findings suggest that public expenditures on long-term care and the healthcare system should continue and/or be augmented to prevent women economic deprivation, especially those who assume caregiving role in their early life. Moreover, the policies and conditions of dual-breadwinner role countries are informative in helping women achieve gender equality.

